# Psychoanalysis and Neuroscience: The Bridge Between Mind and Brain

**DOI:** 10.3389/fpsyg.2019.01983

**Published:** 2019-08-28

**Authors:** Filippo Cieri, Roberto Esposito

**Affiliations:** ^1^Department of Neurology, Cleveland Clinic Lou Ruvo Center for Brain Health, Las Vegas, NV, United States; ^2^Department of Radiology, Azienda Ospedaliera Ospedali Riuniti Marche Nord, Pesaro, Italy

**Keywords:** psychoanalysis, neuroscience, free energy principle, resting state network, default mode network

## Abstract

In 1895 in the Project for a Scientific Psychology, Freud tried to integrate psychology and neurology in order to develop a neuroscientific psychology. Since 1880, Freud made no distinction between psychology and physiology. His papers from the end of the 1880s to 1890 were very clear on this scientific overlap: as with many of his contemporaries, Freud thought about psychology essentially as the physiology of the brain. Years later he had to surrender, realizing a technological delay, not capable of pursuing its ambitious aim, and until that moment psychoanalysis would have to use its more suitable clinical method. Also, he seemed skeptical about phrenology drift, typical of that time, in which any psychological function needed to be located in its neuroanatomical area. He could not see the progresses of neuroscience and its fruitful dialogue with psychoanalysis, which occurred also thanks to the improvements in the field of neuroimaging, which has made possible a remarkable advance in the knowledge of the mind-brain system and a better observation of the psychoanalytical theories. After years of investigations, deriving from research and clinical work of the last century, the discovery of neural networks, together with the free energy principle, we are observing under a new light psychodynamic neuroscience in its exploration of the mind-brain system. In this manuscript, we summarize the important developments of psychodynamic neuroscience, with particular regard to the free energy principle, the resting state networks, especially the Default Mode Network in its link with the Self, emphasizing our view of a bridge between psychoanalysis and neuroscience. Finally, we suggest a discussion by approaching the concept of Alpha Function, proposed by the psychoanalyst Wilfred Ruprecht Bion, continuing the association with neuroscience.

*The real difference lies rather in the fact that the kind and direction of the physical vectors in Aristotelian dynamics are completely determined in advance by the nature of the object concerned. In modern physics, on the contrary, the existence of a physical vector always depends upon the mutual relations of several physical facts, especially upon the relation of the object to its environment*.

Levin (1935), p. 35.

## Introduction

Cognitive neuroscience has made remarkable advances also thanks to the progresses in neuroimaging techniques, such as Positron Emission Tomography (PET) and functional Magnetic Resonance Imaging (fMRI). One of the most important aims of this discipline is the understanding of human brain function. The dialogue between cognitive neurosciences and psychoanalysis is not new, but recently it has become more prolific in the exploration of the relationship between mind and brain, already wished for by Freud more than a century ago and, among others, by the Nobel Prize winner Kandel (1999), when he asserts that psychoanalysis still represents the most coherent and intellectually satisfying view of the mind and can help neurobiologists to plan their work.

To date neurosciences do not provide a consistent, consensual and comprehensive theory about the human brain-mind function, however it is a paramount tool in order to investigate structures and functions about mind-brain in its physiological and pathological development. Psychoanalysis flourished more than a century ago, but despite the first enthusiasm derived from initial fruitful dialogue with neuroscience, we are rather far from understanding the biological basis for all psychoanalytic theoretical frameworks, and this should not be the common goal of psychoanalysts or neuroscientists in their daily work. Although neuroscience and psychoanalysis share the same scientific object of interest, meant as a knowledge in-depth analysis about the functioning of mind-brain system, they use different tools of investigation, different methods and different languages, which requires a separation and distinction, albeit within an ongoing and steady dialogue between the two fields.

Since the birth of psychoanalysis, Freud has attempted to maintain a focus on the neurophysiological phenomena underlying the psychic processes observed. He had to abdicate the pursuit of his dream, first because the technologies available at his time were not sufficiently advanced to seek his neuroscientific ambition and on the other side because of his skepticism about the widespread phrenologic view and the disposition to fit any mental process in its specific brain region. This typical localizationist view was back in vogue after the important Paul Broca’s discoveries in 1861, about the areas of language and his homonymous aphasia, determined by the lesion of an area that still maintains today Broca’s name. Interest was renewed but not completely new, given that since the beginning of the 19th century Franz Joseph Gall, pioneer in the study of the cerebral cortex, focused his neuroanatomical investigation on the attribution of specific psychic functions to specific brain structures. In the first phrenology view Gall believed that man’s moral and intellectual faculties were innate and strictly connected to the organization of the brain. He also proposed a localizationism view of the brain where single regions were responsible for a given mental faculty, and he finally suggested that the development of mental faculties in an individual would lead to a growth or larger development in the sub-region responsible for them.

The phrenology with Gall and the Broca’s localizationism were both a kind of view, an attitude which never satisfied Freud, skeptical about the possibility of embedding every single mental function in its own presumed brain region, frustrated by a static and essentially mechanistic approach, immediately aware about the simplistic, reductive and reductionist imprint of the method. On the contrary he was starting to develop an increasingly dynamic vision of mind and brain.

The old reduction of mental functions to brain structures still finds today numerous supporters and attempts. As Tretter and Löffler-Stastka (2018) pointed out, this attempt encompasses a lot of well-known epistemological, methodological, and conceptual inconsistencies (Block, 1980; Chalmers, 1996; Craver, 2007).

After his experience at the Salpêtriere Hospital in Paris, Freud began to think about large brain networks with a variety of functions, with mutual activation and inhibition properties. An inference that anticipated the concept of *neural networks*, as large brain areas able to activate and inhibit, depending on the activity performed. During the same period, he matured the idea that in the brain there were no isolated centers, or autonomous functions, but instead systems responsible for complex cognitive purposes, composed by several regions, able to be modified by experience.

This dialectic reflects modern day distinctions between functional segregation (or specialization) and integration that have dominated thinking about modern brain imaging. In other words, does one understand distributed processing in terms of specialized regions or the integration and coordination of neuronal activity across brain hierarchies?

Freud’s concept of large brain networks – against the localizationism and reductionist view – showed to anticipate a road that would lead to the concept of complex functional systems, developed more than 50 years later by Lurija (1976), founder of neuropsychology, whose central aim was to reject the idea of reductionism in psychology.

In his Project (Freud, 1895/1963, 1895/1966), Freud tried to explain at various levels the physiological basis of memory, hypothesizing that one of the neurophysiological prerequisites necessary for this function was a system of barriers, which he named “contact-barrier.” He used this term to describe the neurophysiological entity which 11 years later, Charles Scott Sherrington named synapses.

The anticipation of wide and widespread systems dedicated to the realization of cognitive purposes, which today we know as neural networks, becomes impressive within the parallelism between the functions of the ego and specific neural networks, particularly Default Mode Network (DMN), one of the most studied brain networks by the neuroscientific community. Raichle and colleagues coined the term “Default Mode” in 2001 (Raichle et al., 2001); they used PET and described a specific brain state of “rest,” a concept intended to quickly become fundamental in the study of the brain. DMN’s functions seem to play the same mediation function attributed by Freud to the ego (Carhart-Harris and Friston, 2010). In particular, within the DMN, specific regions seem to support the monitoring phases regarding psychological state (Phan et al., 2002), considered areas in which the internal stimuli (bodily and proprioceptive sensations) and inputs from the external environment (e.g., visual and auditory) converge for their integration and development. We will discuss this specific aspect further later.

In the last 10 years, the free energy principle has become *the royal road* in the dialogue between neuroscience and psychoanalysis, *the bridge* between mind and brain. It is linked to the work of Friston and colleagues (Friston et al., 2006; Friston, 2010) and it describes the function of the mind-brain system as any other adaptive biological system, connecting psychological sciences, neurosciences and related fields in perfect confluence and synergy with psychoanalytic concepts (Hopkins, 2012). This approach shows many similarities with typical psychoanalytical concepts as the secondary principle of mental functioning (Carhart-Harris and Friston, 2010), unconsciousness and motivation (Hopkins, 2012), complexity of emotions in attachment (Hopkins, 2015), wish fulfillment within dreaming (Hopkins, 2016), quantitative approach for a formulation of conflict (Hopkins, 2016), and the energic theory within psychoanalysis (Connolly, 2016).

The free energy principle considers the brain as a hierarchical, inferential, Helmholtzian machine, where large-scale intrinsic networks occupy supraordinate levels of hierarchical brain systems that try to optimize their representation of the sensorium, minimizing the amount of free energy (Friston et al., 2006). It represents a process formally close to Freudian metapsychology, in which Freud distinguished two ways of mental functioning: the primary and the secondary processes, corresponding to the pleasure and reality principle, respectively. According to the free energy principle the Bayesian brain uses the Bayesian probability approach to formulate perception as a constructive process based on internal or generative models (Knill and Pouget, 2004; Friston et al., 2006). The brain, with its personal model of the world (von Helmholtz, 1962; Gregory, 1980), tries to optimize this model using new information coming from sensory inputs (Ballard et al., 1983; Friston, 2005). These Bayesian formulations represent a fundamental advance over earlier formulations of optimization in the brain that inherit from behaviorism (e.g., reinforcement learning and optimal control) by explicitly considering (Bayesian) beliefs. In other words, the imperatives for neuronal message passing are framed in terms of belief updating. In this setting, the free energy functional that underwrites active inference under the FEP is actually a functional (i.e. function of a function) of probabilistic beliefs. This is important because it furnishes a calculus of beliefs that is much easier to relate to psychoanalytic constructs (relative to cost or value functions used in behaviorism).

At Freud’s time, one of the most important mental disorders was hysteria, quite widespread among the population at the end of the 1800s. Within this syndrome, neurologists, psychiatrists, psychologists and psychoanalysts – mostly under the debate of the schools of Salpetriêre and Nancy and their leaders Charcot and Bernheim, respectively – were especially interested about the link between mind and body. Among these scholars there was a young Freud as well, who tended to attribute a central role to the body in its connection to the mind. In his Studies on Hysteria (Freud, 1895/1963, 1895/1966), he observed somatic symptoms associated with mental disorders, underlying a close *psychosomatic* connection, after which he elaborated the concept of drive (Instincts and their Vicissitudes, Freud, 1915). With his second topical, in the ego and the id, the psychoanalytic meaning of the body assumes even greater centrality: “the ego is first and foremost bodily entity” (Freud, 1923). Freud thought of the ego as an entity derived from bodily sensations, especially from the sensations coming from the surface of the body. Over the years and deepening of clinical practice, psychoanalysis has begun to configure the link between body and mind not only as fundamental in structuring the ego and with a key role in the relationship with reality, but also with a vision of greater continuity and dynamic fluidity between organic and psychic dimensions, in which the free energy principle represents a useful bridge for the comprehension and communication between neuroscience and psychoanalysis.

Bion (1959) elaborated Freud’s writing “Formulations on the Two Principles of Mental Functioning” (Freud, 1911), particularly focusing his observation on the body and sensory organs as instruments of access to the perception of reality. Bion considered thought and emotion as inseparable components, underlying the central role of the body as the start for the thought phenomena. He focused his observation on sense organs as instruments of access to the perception of reality, explaining how thought is a direct evolution of body sensations. Bion reversed the traditional philosophical conception in which mind produces thoughts: in his theory, there are first thoughts, and mind arises to think them. In other words, mind-body unit is constituted by the body that is in contact with external reality, then there are internal and external sensations, their perception and elaboration that generate emotions, moods and finally thoughts that we eventually perceive as products of the mind (Ciocca, 2015). All this process is supported by α-function. The capacity to transform the sense impressions related to an emotional experience, into α-elements is described as continuous in both sleeping and waking states (Mellor, 2018).

Bion builds a unique model of mind functioning, where the mind faces up continuously to new experiences that cause an emotional impact (positive or negative), and he proposes a general model of functioning of mind in which mental growth depends on the ability of the mind to digest new experiences^1^.

In our manuscript, we try to move through development of a systemic view of the mind, taking in considerations of psychoanalytic models from Freud to Bion, their connections with modern neuroscience and neural networks, underlying the role of the Free Energy Principle as a bridge between mind and brain. We try to assume a methodological parallelism as it was seen for instance by the founder of General Systems Theory, Ludwig von Bertalanffy (von Bertalanffy, 1967), in which a systemic non-reductive multi-level approach might offer better options for integration (Miller, 1978; Tretter and Löffler-Stastka, 2018).

## Psychoanalysis and Free Energy Principle

Despite the extraordinary progresses made by psychology and cognitive neurosciences – among others the deepening of memory functioning and neuroimaging methods – there are few global theories regarding the operating of mind-brain, and no generalized or complete agreement in the neuroscientific community, not even with regard to the meaning of consciousness and unconscious. The proposal of the free energy principle (FEP henceforth) for adaptive systems provides a unified theory of action, perception, and learning (Friston, 2009).

According to Friston (2009), the FEP argues that any self-organizing system in nonequilibrium steady-state with its environment must minimize its free energy, describing how adaptive systems (as biological organisms) resist a natural tendency to disorder (Ashby, 1947; Kauffman, 1993; Friston, 2009). The defining characteristic of biological systems is their attempt to maintain a state of balance toward the constant changes in the environment (Ashby, 1947; Kauffman, 1993), as any homeostatic principle. In the allostatic principle proposed by Sterling and Eyer in 1988, also called a major revision (McEwen, 2004), replacement (Sterling, 2004) of the classical theory of homeostasis, the brain is identified as the central mediator of ongoing system-wide physiological adjustment to environmental challenge (Sterling and Eyer, 1988; McEwen, 2007). Both homeostasis and allostasis are endogenous systems engaged in maintaining an internal balance of the organism, coping with the continuous internal and external changes.

FEP rests on the idea that all biological systems instantiate a hierarchical generative model of the world that implicitly minimizes its sensory entropy by minimizing the level of its free energy (Ramstead et al., 2018). In other words, self-organizing systems, including human being as an example of biological organism, must resist the distributed effects of a natural increase in entropy for their existence, development, and evolution by trying to minimize free energy.

According to Friston et al. (2015a), these self-organizing systems must have a specific identifiable boundary condition: the so-called Markov blanket, which acts as a protective screen, described by Friston et al. (2015a) as a veil through which we are able to recognize and distinguish an internal side from an external environment of an organism, inferring the external or internal causes of sensations, perceptions, or changes. The Markov blanket is not only a protective screen thanks to which we can infer the external causes of the sensorium, it is also operates as a “projection screen” onto which sensory impressions are cast – that are actively solicited by habitual mechanisms (i.e., reflexes mediated by active states), which are used to make sense of the world (Friston et al., 2015a). As any other screen, the Markov blanket allows the separation of an internal dimension from an external environment of an organism, as the case of the cell, which typically represents an immediate and primordial example of a living system with a Markov blanket (Kirchhoff et al., 2018; Mellor, 2018). In this view, the boundaries of a neuron are defined by the external cell membrane, called *plasmalemma*, which is the Markov blanket of the neuron, ensuring separation and identification between an external environment and an internal state, protecting the cell from the external environment, guaranteeing its functions also through this distinction of environments and different electrical charges.

As Connolly (2018) points out, the Freudian energic theory has been widely critiqued by some authors, such as the lack of empirical evidence from neuroscience of the energic processes as described in “The Project” (Zepf, 2010), or the well-known critique by Rapaport (1960), which underlined the impossibility of direct energic processes observation in the clinical situation (Connolly, 2018). Although we cannot observe directly the energy and measure it during the clinical situation (the usefulness of which would be rather limited in any case), we can see the implicit or explicit physiological and psychological attempts by the patients to avoid surprises, especially with non-psychotic patients, who often seem to “prefer and choose” unpleasant and/or painful states (e.g., repetitive, anxious, depressive), but perfectly known, compared to a choice of a change, which apparently could bring emotional, personal, social, or professional benefits, but includes an unavoidable change, surprise, novelty, a new unknown and therefore strongly aversive state. These attempts are definitely psychological and physiological, thus of physical nature.

The mind-brain system tries to maintain the states within physiological bounds, which means trying to maintain a condition where the chances of surprise are minimized, ensuring that internal states remain within physiological and acceptable bounds for the organism. These kinds of attempts are often steady and strenuous, and they are felt as real imperatives in clinical, psychotherapeutic/psychoanalytic settings, in which patients try to avoid surprises and novelties often might be represented by a change of job, partner, or change of any other current distressing situation. A clinical frame experienced as painful by a patient who feels stuck but somehow safer in the current situation experienced as suffering but known, and for this reason is “preferred” to a new one that is potentially and surprisingly dangerous. During this clinical moment it is possible to observe the individual’s effort engaged in his *data assimilation* from the environment, comparing it with the internal data and reality, trying to keep down the entropy levels and minimizing the possibility of surprise, avoiding excessive energy investment in an extremely hard and tiring psychophysical work.

As we mentioned, in the Bayesian brain principle the brain acts having a model of the world (von Helmholtz, 1962; Gregory, 1980), working through active inference, as an inference machine, generating actively predictions (von Helmholtz, 1962; Gregory, 1980; Dayan et al., 1995; Friston, 2010), and with this principle the brain tries to resist to a natural tendency to disorder, maintaining a sustained and homoeostatic exchange with its environment. According to Friston (2010), the brain’s attempt to minimize the variations of free energy (maximizing Bayesian model evidence) not only provides a principled explanation for perceptual (Bayesian) inference in the brain but can also explain action and behavior (Ortega and Braun, 2010). Helmholtz’s model about the brain as an inference machine (Helmholtz, 1866/1962; Dayan et al., 1995) remains a key concept in neurobiology (Gregory, 1980) and psychology.

In this framework the brain works continuously trying to find pattern – thereby reducing free energy and minimizing surprise^2^ – an effort that tries to reduce the free energy, minimizing the surprise from the system. This effort for the most part takes place in a completely implicit, unconscious way. For the individual, surprise means high level of free energy, leading the system to possible incorrect, erroneous, and unreliable predictions in relation to the world around it (Friston et al., 2015b). In psychoanalysis, this inaccurate prediction is translated as a poor testing of reality. Optimal reality testing would therefore require a minimization or reduction in free energy (surprise). This is implicit in belief updating that converts prior beliefs into posterior beliefs that minimize free energy. This can be thought of as the mathematical image of “binding energy” in a Freudian sense, where this “binding” occurs within the boundary established by the Markov blanket (Friston et al., 2015b).

The link between free energy and complexity is straightforward: free energy or surprise can be decomposed into complexity minus accuracy. This means that minimizing surprise (or maximizing model evidence) entails a maximization of accuracy in terms of explaining sensory impressions while, at the same time, minimizing complexity. This corresponds to Occam’s principle and says that we try to find the simplest possible explanations that provide an accurate account of our sensorium.

According to Hopkins (2016), the FEP allows one to observe how the statistical conception of complexity employed by Friston and colleagues relates to emotional conflict and trauma; how symptoms as well as dreams can be understood in terms of complexity-reduction; how in a similar way REM dreaming reduces complexity though the consolidation/reconsolidation of memory; and how complexity and the mechanisms that have evolved to reduce it seem to play a key role for the understanding of mental disorders.

FEP today has a fundamental role in the dialogue with neurosciences and within psychoanalysis itself, describing an important model in understanding and deepening the functioning of the mind-brain system, offering a bridge between neural and psychological processes. As pointed out by Hopkins (2016), this linking of complexity, dreaming, and disorder also indicates that Freud and free association offer a clear and sharp path with cognitive science, free energy neuroscience, and computational psychiatry in order to create a consistent and solid connection between the psychological and neuroscientific views (Hopkins, 2016).

Thanks to the dialogue with neuroscience and the FEP, Freud’s free energy can be related to the potentially unifying paradigm advanced by Friston and colleagues, giving us the opportunity to better understand the mind-brain system in functional and dysfunctional disposition, through the investigation of psychoanalytic theory and models.

## Resting State Networks and the Default Self

In the Ego and the Id (Freud, 1923), Freud claims that the ego is not master in its own house, in other words the conscious instance is neither the only responsible nor the most important factor for the human behavior. The ego is influenced by the contradictory impulses of other instances, whose actions are often hidden. These other instances are the id, present at birth, established by constitution, consisting of impulses and instincts that originate from the bodily organization, finding expression in a psychic unknown form. The other instance, to which the ego is exposed, originates from the internalization of behavior codes, injunctions, social prohibitions felt as constraint and impediment to the enjoyment of satisfaction, a censorship system that regulates the passage by the instinct from the id to the ego. It is a kind of moral censor able to judge human instinctive acts and desires, based mainly on models of value that the child brings from his relationship with parents, often almost completely unconsciously. Freud named this instance superego.

The concept of psychic function elaborated by Freud seems to be consistent with the latest physiological results on the functional organization of the cerebral cortex. The ego is a mental structure characterized by the function of mediating between the inner world, pulses, impulses, desires from the id, prohibitions from superego and stimuli of external reality by ensuring integration and continuity of the individual. This operating entity identified by Freud finds numerous points of contact today with recent studies coming from the resting state networks of the brain.

As with Freud, many other scientists have tried to explain the organization of thinking apparatus with different theories. The father of American psychology, James (1890), proposed the idea of *stream of consciousness*, underlying how the daily life mental activity flows smoothly with or without the presence of specific stimuli from the external environment. During this state of consciousness, the individual is engaged in recording all the information-bodily sensations (somesthesic and vegetative), experiencing free association of stimuli such as thoughts, memories, past experience, inner dialogue, mental images, emotions, day dreaming, planning future events, and other activities. In this state the mind jumps from one thought to another with fluidity and usually with readiness (Cieri and Esposito, 2018). This state of mind, nowadays called Random Episodic Silent Thinking (REST; Andreasen et al., 1995), emphasizes the free and errant nature of this way of thinking, partly in contrast with the engagement of mind during cognitive tasks.

Among modern neuroimaging techniques, today fMRI and Magnetoencephalography (MEG) allow for the study of the brain *in vivo*, opening the intersection of anatomy and functions (Cieri and Esposito, 2018). fMRI can be performed during the execution of an experimental paradigm involving specific cognitive tasks or to study spontaneous oscillations of brain activity while the REST of the subject (resting-state fMRI, rs-fMRI; Raichle et al., 2001; Buckner et al., 2008; Cieri and Esposito, 2018; Esposito et al., 2018a). Since it does not require any task, rs-fMRI is characterized as particularly suitable for studies on subjects such as children and elders, because this protocol does not require any particular skill or specific attention focus from the subject, increasing the compliance of the participant and reducing intersubjective variability due to the task performance (Esposito et al., 2018a). Indeed, in recent years a growing number of studies showed that rs-fMRI could be considered as an additional important tool for the investigation of physiological and pathological mental conditions.

Spontaneous brain activity generated in absence of cognitive task has been discussed in the last two decades, representing a pivotal role among psychological and cognitive neuroscientific fields. Many neuroimaging studies considered this brain activity a functioning model of the mind. Cerebral activity recorded during cognitive tasks showed a baseline low frequency fluctuation (0.01–0.1 Hz). In this light some researchers examined these cerebral baseline activities based on the idea that those low levels of brain activity could represent real active states, and that brain activation patterns represent a shift in focus from an internal self-referential state to an external focus (Raichle et al., 2001). This discovery encouraged neuroscientists to begin to consider two different types of neuronal activity: evoked and spontaneous (Fox and Raichle, 2007; Barrett and Simmons, 2015). Brain spontaneous activity has received growing attention (Buckner et al., 2008) in the last decade, supported by several studies showing electric activity, hemodynamic and metabolic parameters, spontaneous fluctuations of membrane potential, spontaneous spikes and neurotransmitter release (O’Donnell and van Rossum, 2014).

During the early 21st century, several studies using PET (Shulman et al., 1997) and task-fMRI (Gusnard and Raichle, 2001) identified specific regions active during cognitive task execution and other brain areas active during different REST conditions. These latter neural regions constituted a network involving both hemispheres: the Medial Prefrontal Cortex (MPFC), the Posterior Cingulate Cortex (PCC), the Inferior Parietal Lobules (IPL), and Hippocampal Regions (HP), forming the neural network called DMN, engaged when mental activity is internally directed, when an individual is left “undisturbed” to think about himself, wondering about his life, his past or future. One hypothesis about the DMN’s functioning concerns its involvement in inner mental processes far from all external stimuli, building dynamic mental simulations based on past personal experiences used in recalling memories; it also supports the mental process about the future, and generally when an individual imagines alternative scenarios to the present (Buckner, 2013). This network is also known as a task-negative network because its regions are typically deactivated during execution of attention demanding tasks (Passow et al., 2015).

For many years, modern neuroimaging techniques neglected this important spontaneous activity of the brain, focusing only on changes evoked by external cognitive tasks. During the last two decades, rs-fMRI has become a most utilized tool to study the brain *in vivo*, especially for those patients less cooperative as we mentioned, offering detailed and clear information about the spontaneous brain dynamics in both physiological and pathological conditions (Cieri and Esposito, 2018). Indeed, one of the most important common aims of neuroscience is to identify early biomarkers in order to reach an early diagnosis, providing a timely and specific treatment, even if today we are far from understanding, the neurobiological or neuropsychological markers of all neurological or neuropsychiatric conditions. An important step for neuroscientists and psychoanalysts, useful to reach the mentioned aim to identify early biomarkers, is linked to the deepening of the relationship between mind and brain and mind and body communication. According to Solms (2019), adopting a dual-aspect monist position on the philosophical mind-body problem allows one to find the causal mechanism of consciousness not in the manifest brain but rather in its functional organization, which ultimately underpins both the physiological and the psychological manifestations of experience. Adopting a dual-aspect monist position, using neuroimaging techniques and approaches such as FEP will allow psychoanalysis and neuroscience to investigate this functional organization, studying in deep analysis the mechanisms underlying physiological or pathological human conditions. In this sense and with this common aim, the resting state networks together with the FEP could play a key role in the study of the mind-brain system.

Within this dialogue, the DMN seems to play the same function of mediation attributed by Freud to the ego, and some authors have spoken about Default Self (Beer, 2007; Qin and Northoff, 2011) in order to define the DMN as a kind of biomarker of the Self. Nevertheless, the experience in the perception of the Self is extremely complex, characterized by high variability, and it is not always easy and clear to distinguish the Self from all other phenomena related to cognitive processes. In any case, the role of DMN within the functions of the Self is conspicuous as shown from several psychopathological studies, where the impairment of DMN connectivity associated with an impairment of Self’s experience is noticeable.

## Resting State Networks in Neuropsychiatry

In recent years, there has been a growing interest about abnormal functional connectivity in neurologic and neuropsychiatric disorders, although the results remain debatable. For instance, despite still controversial claims, DMN shows anticorrelated activity with another REST network, the Dorsal Attention Network (DAN), conversely active during externally-directed cognition, such as cognitive tasks that require conscious and focused attention. This anticorrelation could be impaired in some neurological conditions such as Mild Cognitive Impairment (MCI – Esposito et al., 2018a).

Although the focus of this article is not about the use of resting state functional connectivity to assess brain circuits in psychiatric or neurological disorders, it is certainly useful to underline some important issues and connections. Abnormal functional connectivity could be found both in studies on neurodegenerative and neuropsychiatric disorders, including anxiety, major depressive disorder (MDD), bipolar disorder (BD), obsessive compulsive disorder (OCD), schizophrenia (SZ), attention deficit hyperactivity disorder (ADHD), autism spectrum disorder (ASD), Eating Behavior Disorder (EBD), Alzheimer’s disease (AD), and other neurodegenerative disorders (Buckner, 2013; Andrews-Hanna et al., 2014; Cieri and Esposito, 2018; Esposito et al., 2018a).

In the physiological aging process, the integrity of the DMN is diminished both in function (Andrews-Hanna et al., 2007; Cieri and Esposito, 2018) and structure (Turner and Spreng, 2015), and these changes are associated with MCI, especially in memory functions. Moreover, social cognitive impairments in aging have been associated with reductions in activity within the Dorso Medial Prefrontal Cortex (DMPFC; Moran et al., 2013). These impairments increase in the dimensions of pathological aging as AD and forms of frontotemporal lobar degeneration (FTLD), including semantic dementia (SD) and behavioral variant frontotemporal dementia (bvFTD – Andrews-Hanna et al., 2014).

Schizophrenic patients have shown a dysfunction of an important area of DMN, the Anterior Cingulate Cortex (ACC) associated with difficulty of recognizing actions and functions, correlating with their positive symptoms (Carter et al., 2001). ACC reduces its activity during external cognitive stimulation, highlighting its fundamental role in self-referential mental activity, in close relation with another important region in this process: the anterior insula (AI). Coactivation of these two areas might play a key role in establishing the self-functions (Esposito et al., 2018a,b). In schizophrenic patients, we can observe the typical lack of symbolic ability, lack of activity to make predictions, and mental simulations, often accompanied by the absence of dream activity.

As Andrews-Hanna et al. (2014) pointed out, both the nature and topographical locations of DMN alterations differ across disorders, paralleling varied symptom profiles. While disorders of integrity (e.g., AD) are often associated with hypo-activation or connectivity of a particular DMN component and impairments in specific aspects of self-generated cognition, disorders of content (e.g., depression) and regulation (e.g., ADHD) are typically associated with hyperactivation and hyperconnectivity, paralleled by polarized or excessive forms of self-generated thought (Andrews-Hanna et al., 2014). Moreover, the body image disturbance in EBD may be supported by a modification in connectivity within specific cortical areas like the precuneus (PrC; Seojung et al., 2014), and this result could represent the neural correlates underlying increased self-focus, rumination, and cognitive control in relation to eating disorders and the impairment about the body perception (Esposito et al., 2018a,b).

Within the DMN, specific regions support the self-reported mental processes, monitoring psychological states (Phan et al., 2002) and could be considered regions of convergence receiving internal (bodily and proprioceptive sensations) and external inputs (visual and auditory), for their integration and development. These areas are the cortical midline regions and among these regions the most important are MPFC and ACC, associated with the control of various functions such as selecting or inhibition of some response, monitoring the conflict and identification of errors (Schneider et al., 2008). ACC plays a fundamental role in affective evaluation (Allman et al., 2001), conflict monitoring and detection (Botvinick et al., 2004), response selection (Awh and Jonides, 2001), and attentional control (Posner, 1994).

Andrews-Hanna (2012) and Buckner (2013) hypothesize that one of the major functions of the DMN, perhaps the most important, is to support internal mental simulations used adaptively. This concept is consistent with FEP in which the system is engaged with its simulations, searching of patterns, trying to maintain an internal sensitive balance of the organism, supporting internal mental simulations used in an adaptive way. In other words, the research of patterns claimed by the FEP is consistent with the DMN’s most important role, mediating between the external and internal stimuli, building dynamic mental simulations based on past personal experiences used in recalling memories. This is the same function attributed by Freud to the ego (Carhart-Harris and Friston, 2010).

The “investment” of the system in energy terms, in trying to keep lower levels of entropy, decreases the chances of having to face surprises or when optimally attuned to the world, seek out novel situations that will minimize surprise in the future (i.e., expected surprise or uncertainty).

Many neuropsychiatric and neurological diseases are characterized by the impairment or lack of important symbolic function, strictly linked to the Self and to the ability of the system to support internal mental simulations used in adaptive way. Recent findings suggest the existence of a frontoparietal control system consisting of flexible hubs that regulate distributed systems (e.g., visual, limbic, motor) according to current task goals (Cole et al., 2014; Cieri et al., 2017).

DMN seems to directly contribute to all inner mental processes supported by the MPFC and its links to the HP, with its known key role in memory functions. To support this complex interaction, DMN is constituted by two subsystems. The first is the temporal-mesial subsystem, associated with mnemonic processes, activated during retrieval of past memory; this subsystem is predominantly made up of HP and shows high connectivity with another two important brain regions typically active during memory tasks: PCC/PrC (Posterior Cingulate Cortex/Precuneus) and IPL. The second subsystem is connected to the MPFC, specifically dorsal-MPFC activated during mental situations of self-exploration and sensations. The results suggest that self-referential mental activity engages a preferential MPFC subsystem (Szpunar et al., 2007). These functions are closely related to DMN anatomy: two interactive subsystems whose predominant areas are HP and MPFC that converge on the retrosplenial cortex (PCC/PrC).

In the last two decades, rs-fMRI studies have allowed the identification of a set of different networks, not only the DMN, identified in a series of resting-state functional connectivity studies (Greicius et al., 2003; Fransson, 2005; Fox et al., 2006). In fact, besides the DMN, at least 10 RSN networks have been consistently described in healthy populations (Mantini et al., 2009; van den Heuvel and Hulshoff Pol, 2010; Deco et al., 2011), highlighting that the human brain has a network-based organization at REST. Of these 10, the most studied include the DMN, the Salience Network (SN), the Control Executive Network (CEN) (lateralized in both hemispheres), the primary Sensory Motor Network (SMN), the Extrastriate Visual System (EsV), and the DAN (Deco and Corbetta, 2011).

Important to note in this context is the DAN and its specific behavior related to DMN and the Self. DAN includes Inferior Parietal Sulcus (IPS), Frontal Eye Field (FEF), ACC, and bilateral Middle Temporal Gyrus (MidTempG), and it has received much attention because – conversely to DMN – it is called the task-positive network, being active during cognitive tasks which demand attention and mental control (Corbetta and Shulman, 2002; Fox et al., 2006; Esposito et al., 2018a,b). DMN and DAN show a pattern of anticorrelation in their activity in both task and resting state studies, suggesting that they are intrinsically organized into anticorrelated networks (Fransson, 2005; Esposito et al., 2018a,b). This DAN-DMN anticorrelation during resting state may represent a cerebral mechanism supporting cognitive functions (Gopinath et al., 2015), switching focus between internal, supported by DMN, and external channels and attention demanding events, supported by DAN (Esposito et al., 2018a,b). Interestingly, this negative correlation between DAN and DMN modifies its function during life span. In fact, consistently with function and evolution of the Self, it appears during the first year and it strengthens during the second year of life (Barber et al., 2013).

As mentioned, the concept of Self cannot be seen as a static and steady entity, but rather dynamic in its development and evolution. Developmental psychology claims that a first concept of Self flourishes between the first and second year of life, when the child begins to recognize himself as an object. According to Craig (2011), the most important sign of self-awareness is the ability of the child to recognize himself in the mirror. In parallel, with the growth of the individual Self, the negative correlation between DAN and DMN becomes stronger in adults to support the development of executive functions and working memory from childhood to adulthood (Andrews-Hanna et al., 2007; Cieri and Esposito, 2018).

Following this process, a decreased anticorrelation between these two networks starts to appear weaker during physiological aging (Wu et al., 2011), increasing its weakness in the case of MCI (Esposito et al., 2018a,b), representing a possible biomarker of neuroaging, cognitive decline, and first impairment of self-functions.

## DMN and Freudian Secondary Principle

Carhart-Harris and Friston (2010) proposed the consistency of the Freudian concept of secondary process with the DMN functions, capable of self-organizing and suppressing free energy, such as the anarchic and unconstrained endogenous activity from the limbic and paralimbic systems. The mind-brain system tries to maintain its state within physiological bounds, trying to minimize the possibility of surprise. This constant attempt to avoid surprise, ensuring that the states remain within physiological bounds, is consistent with the neurophysiological functions of the brain, specifically with the function of DMN.

According to Carhart-Harris and Friston (2010), the construct validity of Freud’s hierarchical organization of the mind, with its distinction between id and ego – belonging to the primary and secondary processes, respectively – can be enhanced by remarkable consistency with contemporary models of cognition based on hierarchical Bayesian inference and Helmholtzian free energy. In fact, Freudian metapsychology distinguished two ways of mental functioning, the primary and the secondary processes, corresponding to the pleasure and reality principle, respectively. The primary process is driven by the pleasure principle, which is in turn driven by the id and its instinctual functioning with its instincts and desires, without taking into account the constraints of the external environment with its rules and laws. The secondary process, also called the reality principle, is governed by the ego, which controls the instant gratification mentality of the id. The reality principle is the ability of the mind to assess the reality of external world and to act accordingly with it, as opposed to the pleasure principle.

Freud studied the function of the mind through these different processes, as two fundamentally different styles of cognition, also through a study of non-ordinary states of consciousness (e.g., hallucinations and dreams), in which he recognized a mode of cognition characterized by a primitive style of thinking (Carhart-Harris and Friston, 2010). He speculated that in these primitive non-ordinary states of consciousness, the exchanges of neuronal energy are free, and he designated it as the primary process (Freud, 1940). Moreover, in these non-ordinary states, he identified the loss of certain functions, usually present in “normal” waking cognition, ascribing these functions to a central organization of the ego, which works in order to minimize free energy of the mind, underlying the specific property of this function belonging to the secondary process, defining its aim as one of converting free energy into bound energy states (Carhart-Harris and Friston, 2010).

The Freudian concept of reality principle seems consistent with the functional role of the DMN in its hierarchical and self-organizing role of suppressing free energy originated from subordinate levels, such as the limbic and paralimbic systems. In fact, the Freudian secondary process with its top-down mode of operation, in which it transforms free energy of the lower levels into bound energy trying to keep the system on physiologically acceptable levels, seems to be consistent with the functions of the DMN.

Under this mapping between Freudian and Helmholtzian models, is possible to link the energy associated with the primary process and the free energy of Bayesian formulations; in both accounts, higher cortical areas try to organize the activity from the lower-levels through suppression of their free energy (Carhart-Harris and Friston, 2010).

Another important feature of DMN consistent with FEP is the mentioned anticorrelation, the inverse relationship of its neurophysiological activity with DAN (Corbetta and Shulman, 2002; Fransson, 2005; Fox et al., 2006; Esposito et al., 2018a,b). These intrinsic networks correspond to the high-levels of an inferential hierarchy, which function to suppress the free energy of lower levels (i.e. suppress prediction errors with top-down predictions), associating this optimization process with the Freudian secondary process. Also, the failures of top-down control with non-ordinary states of consciousness, such as early and acute psychosis, the temporal-lobe aura, dreaming, and hallucinogenic drug states (Carhart-Harris and Friston, 2010), might be associated with an impairment of the supraordinate system, as DMN is unable to control in a top-down mode the excess of the free energy from the lower system.

Moreover, as we noticed, the DMN functional connectivity seems to become relatively weak in the elderly (Damoiseaux et al., 2006; Andrews-Hanna et al., 2007), representing a neurological impairment of the mechanism able to support cognitive functions, switching the focus from the inside supported by DMN, to the outside supported by DAN. We can observe the higher control system apparently impaired and unable to bind the free energy, making difficult the executions of cognitive tasks. In cases of ADHD (Castellanos et al., 2008) or impulse control disorders (Church et al., 2009), the hierarchically lower system seems to become too active to be managed by the hierarchically superior system, operating a sort of “mutiny,” or “hijacking,” leading to an impairment of the system control.

MPFC-PCC connectivity is entirely absent in infants (Fransson, 2005) and the DMN develops through ontogeny, in a way that runs parallel to the emergence of the individual Self with its complex functions.

The spontaneous fluctuations in neuronal activity from cortical nodes of DMN suppress or contain the unconstrained and anarchic endogenous activity of limbic and paralimbic systems (Helmholtz free energy). This neurobiological view rests on the basis of the brain as a hierarchical, inferential, Helmholtzian machine, in which large-scale intrinsic networks such as the DMN are located at higher levels of cerebral hierarchy and work to optimize the representation of the sensorium, minimizing the level of free energy. As Carhart-Harris and Friston (2010) indicate, this optimization, formulated as minimizing free energy, is similar to the treatment of energy in Freudian formulations, and developing these points of contact may help anchor Freudian concepts to more rigorous biological phenomena, helping not only psychoanalysis but the entire neuroscientific field.

As Solms (2014) specifies, when Friston claims about minimizing prediction error and giving up on predictive models that do not correspond to external states, he is making reference to Freud’s reality principle, while in a different frame of reference. Freud’s descriptions of the secondary process are consistent with the functional anatomy of large-scale intrinsic networks and how this process works to minimize free energy, with its hierarchical organization and continuous and constant attempt trying to keep low levels of surprise. Also, as outlined above, this concordance find is an interesting conceptual hook trough the development of functional connectivity between the nodes of the DMN during ontogeny, as a process that runs parallel to the emergence of the Self’s functions.

Freud always explained clinical phenomena in terms of natural forces and energies, not surprisingly he was a student of Helmholtz’s medical school and in this regard, it is interesting to note that in 1898 Wilhelm Fliess – an otorhinolaryngologist, passionate scholar of psychoanalysis, and close friend of Freud – sent to him two big volumes of Helmoltz’s lessons as a gift in honor of their good friendship and their common attendance and interest in the famous physiologist’s lessons and theories.

In the context of the dialogue between psychoanalysis and neuroscience, it might be beneficial for the neuroscience field to try to find contact points with psychoanalysis in order to nourish an inextricable dialogue, started from the birth of psychoanalysis which can certainly improve, providing benefits to the understanding of the mind-brain system in physiological and pathological conditions.

## Wilfred Ruprecht Bion: The Theory of “Alpha Function”

Freud described the establishment of the principle of reality, underlying how consciousness develops through the perception of the outside world and in addition to the dualism of pleasure-sorrow (the principle of Nirvana – primary narcissism), perception is characterized by manifold sensory qualities (Freud, 1911). Freud’s principle of reality is the ability of the mind to assess the outside world, acting accordingly with it in opposed direction to the principle of pleasure (Freud, 1940). Thought is a substitute for motor discharge, even though the latter never stops functioning as a mechanism to release psyche. The establishment of the principle of reality allows the development of a mental function to defer instant gratification, the governing principle of the actions taken by the ego, after its slow development from a “pleasure-ego” into a “reality-ego” (Freud, 1940).

What Freud defined as attention, a mental function that explores outside world, is consistent with Bion’s alpha function (α-function), theorized in “Learning From Experience” (Bion, 1962a). In the personality, there are several factors that combined with each other form the personality functions, a term with which Bion intends the mental activity (Bion, 1962a,b). Through α-function, non-mental elements (sensory impressions, β-elements) are processed into mental elements (α-elements), giving them an emotional connotation (good, pleasant, unpleasant, and bad). β-elements are the raw material of mental process, impressions of sensory activation, perceptions of internal and external body state changes that have no meaning and are perceived physically. Everything that is emotionally lived must be at first elaborated by the α-function; this implies that emotional experiences, lived both during sleep and wakefulness, must be elaborated by α-function. When a patient is insufficient in α-function, the β-elements are not thinkable and they can fall under projective identification (Acting-Out). In this case, a patient cannot transform sensory impressions into α-elements and therefore cannot dream. In order to learn from experience, α-function must operate on the basis of emotional experience by generating α-elements that will be used by thought that works in the dream and in the unconscious (Bion, 1962a, 1973). Dream and α-function are located between conscious and unconscious, differentiating them through a barrier that Bion calls the *contact barrier* that preserves personality from psychotic state. α-function (both awake and during sleep) transforms sensory impressions linked to a specific emotional experience in α-elements that proliferate and condense, forming the contact barrier. The elements can pass freely through the contact barrier between conscious and unconscious states, and the dreams allow us to access directly the contact barrier (Bion, 1962a; Mellor, 2018).

Psychotic patients do not have α-function, resulting in the inability to transform sensorial impressions into α-elements, to dream and to generate conscious and unconscious. In fact, the contact barrier, with its properties necessary to distinguish mental phenomena (conscious and unconscious), is missing in psychotic patients and replaced by the *beta screen* (β-screen) composed of β-elements. Psychotic patients invert α-function, and sensory impressions are no longer used to form α-elements and the contact barrier. α-elements, contact barrier, unconscious thoughts, and dreams are redirected to β-elements and projected to form the β-screen. The inversion of α-function does not recompose β-elements but creates “*bizarre objects*.” Indeed, β-elements are sensory impression and do not have traces of personality, while bizarre objects have traces of personality (ego and super-ego). α-function, during the transformation of emotional experience into α-elements, plays a key role in the sense of reality and its inactivity produces disastrous effects on personality such as deep psychotic deterioration.

According to Freud, thoughts are born through the absence, while for Bion thoughts are precedent to thinking, and the latter develops for the necessity to treat thoughts. Bion hypothesized that the mind is a container of thoughts and the α-function develops to contain and process thoughts. It is possible to disengage the mind from thoughts by primitive defense mechanisms, such as expulsion (Freud, 1937), if the personality is prepared to avoid frustration. If, on the other hand, personality is dominated by the impulse to bear and change frustration, it will think the thoughts. If the patient is not able to think his own thoughts, he will have an increase in frustration. Bion underlines that the bear of frustration is a genetically pre-established factor of personality. Models of mental functioning are characterized by the inability to tolerate frustration, suffering, anxiety, and the need to use powerful defense mechanisms: splitting, projection, and projective identification. However, Bion adds that the defense mechanisms concern not only emotions and feelings. Indeed, he proposes a psychotic defense mechanism that splits and free ourselves not only of the intolerable affective content, but of the apparatus that allows its perception, a kind of amputation of specific mind functions. Psychotic defense leads to the impoverishment not only of emotions but also of mental abilities.

All the noted Bion’s mind-body unit is in contact with the external reality with the internal sensations supported by α-function. In light of this hypothesis, the mind-body relationship must be seen in continuous dynamism: in harmonic condition, body and mind are integrated with each other, while in disharmonic condition a messy sensoriality that hampers thinking predominates (Ferrari, 1992; Lombardi and Pola, 2010; Lombardi, 2016). In “Transformations” (Bion, 1965), Bion introduces the concept of O. O is the origin as in the geometrical example of the Cartesian axes: experiencing O represents the experience of whole sensations and emotions, which are activated in contact with reality.

As we noted, Francis Joseph Gall (Livianos-Aldana et al., 2007) was the first neuroscientist to study the cerebral cortex, underlying that the brain was made up of several interconnected areas and each of these areas with a specific function. Questioning René Descartes’s theory of mind-body dualism, Gall argued that the brain was the seat of intelligence, a theory that was elaborated only after the development of modern psychology. Thanks to Gall’s theory, mind was no longer considered separate from body, but as an integral part of the organism in its totality. He noted that the brain was the organ delegated to intellectual, moral and affective faculties, identifying higher psychic functions in the frontal cortex. Empirically, through fMRI studies, cortical midline structures and DMN have often been highlighted to be specific for the Self (Qin and Northoff, 2011). DMN is involved in internally oriented self-related processing that comprises surveillance of internal states (emotional, bodily), resulting in what is called “mind wandering” (Mason et al., 2007). Observing resting state networks in their totality, including the subnetworks (Deco et al., 2011) and their interconnection, we may better understand the mind-body unit. Menon (2011) talks about the “Triple Network,” underlying functional interchange between three neural networks: DMN, SN, and CEN. Specifically, DMN with its areas MPFC, PCC, Angular Gyrus, and medial temporal lobe structures plays and important role in monitoring self-referential mental activity; the SN through ACC and Insula, receives and elaborates body sensations and cognitive relevant events engaging frontoparietal systems; CEN, whose key nodes include the dorsolateral prefrontal cortex (DLPFC) and PCC, maintains and elaborates working memory information and decision-making of goal-directed behavior. These networks interact dynamically, mediating cognitive and emotional states (Yu et al., 2018). The SN (Seeley et al., 2007) is involved in bottom-up direction of salience events, involved in detecting, integrating and filtering relevant interoceptive, autonomic and emotional information, and it plays a key role modulating and switching other resting state networks (Menon and Uddin, 2010).

Activation of SN determines an increase of connectivity between DMN and CEN, modulating not only the activation of the networks but also their interconnectivity (Di and Biswal, 2015). SN indeed represents core hubs of the whole brain sending information in other regions and networks. SN through the AI constitutes the hub involved in the registration of internal (body sensations) and external salient events, sending information to DMN that integrates and elaborates information supporting mental activity connected with the Self (Craig, 2010). AI elaborates affective information, pain and empathy, whereas the dorsal part of ACC (dACC) was most closely associated with conflict resolution and cognitive control. The insula and dACC probably constitute a functional circuit involved in interoceptive and affective processes and form an anatomically tightly coupled network ideally placed to integrate information from several brain regions. The insula distributes sensory information coming from the body, in contact with the external reality, and transmits it to further brain regions that allow its processing. In summary, the insula supports emotional experience resulting from bodily states. In line with Bion’s theory, bodily sensations shape emotional experiences, and experiencing O implies the possibility to record the sensations, perceptions and emotions that are activated in contact with reality and therefore experiencing them (Damasio, 1996; Ciocca, 2015). The insula is anatomically situated in a brain area connected with several neural functional circuits supporting cognitive, homeostatic, and affective systems and constitutes a bridge between brain regions involved in monitoring internal states (visceral sensory, somatic sensory processes, autonomic regulation of the gastrointestinal tract and heart; Menon and Uddin, 2010) and that support their processing. The insular cortex registers body sensations and through the interaction with other brain areas, gives rise to emotions that modulate the behavior (Singer et al., 2009; Craig, 2010). Craig and colleagues, in an animal model, identified an ascending pathway from the spinal cord (lamina I neurons in the spinal cord) through the spinothalamic tract, passing through the Nucleus of the Solitary Tract (NTS) and ventromedial nucleus of the thalamus and finally landing at the dorsal insula projecting information to AI and ACC (Critchley and Harrison, 2013). They called this pathway the “*homeostatic afferent pathway*” (Craig, 2009) that carries information about the body. Particularly, information arising from the body reaches the middle and posterior parts of the insula and then is projected in the anterior insula. The awareness of salient events is represented in the anterior insula, whereas more sensory attributes are represented posteriorly (Craig, 2002). The insula represents a core area that receives bodily information, filtering salient stimuli, processing them and then engaging, through ACC, the CEN that supports working memory, higher order cognitive processes and the DMN that supports cognitive functions and Self.

The Freudian description of the mind underlines how bodily experience gives rise to and shapes thought. Pre-reflective representations of visceral states of the Self are linked to activations in the posterior and middle Insula; DMN is engaged when introspection and reflection are needed (Critchley and Harrison, 2013). Interactions between the DMN and insula support the ability to represent one’s bodily states to enable conscious reflection on those states (Molnar-Szakacs and Uddin, 2013). Thoughts derive from integrated physiological activation filtered by the insula and he mind develops to process, contain and give them meaning through DMN.

## Discussion and Conclusion

The dialogue between neuroscience and psychoanalysis is still complex and often conflicting; a controversy deriving foremost from the complexity of the study object: the mind-brain system, perhaps the most complex and challenging subject for the human being, from a scientific, philosophical, and psychological point of view. A second reason, which probably did not favor the discourse between these two disciplines, derives from the conceptual conflict of conceiving a system able to study itself. Both in the case of neuroscience as in the case of psychoanalysis, the subject and the object of the investigation coincide, and this aspect becomes an evident limitation in the study of any phenomenon. Specifically, these two disciplines use different tools and methods, sharing the same target: the knowledge of the mind-brain system, its development, and its physiological and pathological expressions. The differences in methods and tools used have not discouraged and should not discourage at all this fundamental relationship. Instead, the innovative approach of resting state network investigations has facilitated the communication, opening new horizons. Resting state networks in general and DMN in particular opened a window on neurophysiological mechanisms linked to spontaneous thought processes, not exclusively related to the active execution of cognitive tasks. The greater knowledge of neural networks functioning allows a theoretical deepening on spontaneous and unconscious thought processes and in general on mind-brain functioning and on the mind-body relationship. Although the beginnings of modern neuroscience have been characterized by a cognitive psychology approach, with a tendency to exclude affective, emotional and unconscious processes – in which the unconscious was often defined as implicit or unaware – over time thanks to scientific evidence and clinical practice, it was no longer possible to exclude emotional and unconscious states from neuroscientific studies. This point brought neuroscience back to the approach originally conceived by Freud with the investigation through the resting state networks that confirms and deepens this relationship. Progresses made in the field of neuroimaging allow a deeper and more detailed investigation, therefore a greater understanding of psychoanalytic theory, models and observations, and the mind-brain system in its functions and dysfunctions, finding in concepts such as FEP its natural meeting point, its *bridge* between mind and brain, in which Freud’s more speculative free energy theory, based on the clinical method, find a natural connection with the more rigorous methods of neuroscience, a goal to which Freud himself aspired since the birth of psychoanalytic theories.

FEP takes elements from the Bayesian and Helmoltzian approaches, conceiving the human mind as perpetually committed in active inference, analyzing data from the sensorium and from external reality, comparing and analyzing them, trying to keep down the entropy levels and therefore minimize the possibility of surprise (and seeking out opportunities to minimize surprise), thereby avoiding excessive levels of free energy (Friston, 2010). Seth and Friston (2016) recently described active interoceptive inference, providing an interesting and detailed set of concepts within which to conceive the neurofunctional basis of emotion, embodied selfhood and allostatic control. The neuronal activity encodes expectations about the causes of sensory input, where these expectations aim to minimize prediction error and where the prediction error lies in the difference between (ascending) sensory input and (descending) predictions of that input. This minimization rests upon recurrent neuronal interactions between different levels of the cortical hierarchy. For interoceptive inference, predictions issue from visceromotor areas and project to viscerosensory areas (to provide corollary feedback) as well as to brainstem and subcortical areas (to engage autonomic homoeostatic reflexes). The authors point out how visceromotor predictions are best interpreted as providing homoeostatic set-points that enslave autonomic reflexes and guide allostatic (behavioral and physiological) responses *via* interoceptive prediction errors at different hierarchical levels and timescales (Seth and Friston, 2016).

In the FEP the brain acts having a model of the world, working through active inference generating actively predictions to minimize the variations of free energy (maximizing Bayesian model evidence), providing a principled explanation for perceptual inference in the brain. With this principle, the brain tries to resist its natural tendency to disorder, maintaining a sustained and homoeostatic exchange with the environment.

As we mentioned, the DMN is consistent with ego functions and with its target of containing free energy levels of underlying structures, a function of the secondary process. The result is a top-down hierarchy of DMN which aims to reduce the free energy associated with the Freudian primary process. The cortical regions modulate the activity of subcortical areas, ontogenetically and phylogenetically older, through the lowering and optimization of free energy. Freudian constructs of the primary and secondary processes seem to have neurobiological substrates, consistent with self-organized activity in hierarchical cortical systems, and Freudian descriptions of the ego are consistent with the functions described of the DMN with its reciprocal exchanges with subordinate limbic and paralimbic brain systems.

Even in Bion’s theory, the body is in close contact with external reality; internal and external sensations trough α-function shape emotional experiences and finally thoughts. Learning from experience represents the attempt of individuals to experience the emotion of the moment without running away in the knowledge, which would be the result of a defense mechanism aimed at the avoidance of that specific emotional state. The insula, with its connections with several neural functional circuits, supports emotional experience resulting from bodily states.

The anterior insular cortex is a part of the visceromotor area, situated at the top of an interoceptive hierarchy (Seth and Friston, 2016); it receives ascending projections from viscerosensory areas (e.g., posterior and mid-insula) and their descending connections engage a range of subcortical, brainstem, and spinal cord targets involved in visceromotor control, such as the periaqueductal gray and the parabrachial nucleus (Seth and Friston, 2016). The anterior insula constitutes a hub involved in the registration body sensations and filters external salient events, then sending information to the DMN that integrates and elaborates information supporting mental activity connected to the Self. The insula and dACC constitute a functional circuit that integrates information from several brain regions. They form an anatomically tightly coupled network ideally placed to distribute sensory information to further brain regions that allow their processing.

As we noted, Bion (1959) focused his observation on body and sensory organs as instruments of access to the perception of reality, considering thought and emotion inseparable components of the same process, underlying the central role of the body as the start for the thought phenomena. In his theory, digestion of new experiences corresponds to the “data assimilation” or “evidence accumulation” implicit in “belief updating” under the FEP.

Although we do not know if psychoanalysis should help to plan the work of neurobiology, as claimed by Kandel 20 years ago (Kandel, 1999), we believe that a dialogue between these two disciplines should increase in light of new developments, without prejudices in name of curiosity and respect for the history, tools, methodologies, and languages used by the different approaches, in order to reach important advances in the knowledge of the mind-brain system, in which other disciplines as psychiatry, psychology, and neurology could naturally take advantage in order to improve the diagnostic and therapeutic approach to mental suffering.

## Author Contributions

RE and FC equally contributed to the manuscript.

### Conflict of Interest Statement

The authors declare that the research was conducted in the absence of any commercial or financial relationships that could be construed as a potential conflict of interest.

## Footnotes

^1^Technically, the digestion of new experiences corresponds to the “data assimilation” or “evidence accumulation” implicit in “belief updating” under the FEP.

^2^An interesting corollary of surprise minimization is that we are compelled to seek out novelty, because novelty affords the opportunity to reduce expected surprise or uncertainty. One common feature found in non-psychotic patients concerns a certain “extension”, or shift of discomfort from surprise to novelty in general, as if any novelty could lead to a risk of compromising the system. This mechanism is easily found in depressive, anxious or obsessive patients, in which we can observe how they try to avoid and defend themselves from novelty, as well as from surprise, repeating their same known and “safe” patterns.

## References

[ref1000] Allman J. M. Hakeem A. Erwin J. M. Nimchinsky E. Hof P. (2001). The anterior cingulate cortex: the evolution of an interface between emotion and cognition. Ann. N. Y. Acad. Sci. 935, 107–117. 10.1111/(ISSN)1749-6632, PMID: 11411161

[ref1] Andreasen N. C. O’Leary D. S. Cizadlo T. Arndt S. Rezai K. Watkins G. L.. (1995). Remembering the past: two facets of episodic memory explored with positron emission tomography. Am. J. Psychiatry 152, 1576–1585. 10.1176/ajp.152.11.1576, PMID: 7485619

[ref2] Andrews-Hanna J. R. (2012). The brain’s default network and its adaptive role in internal mentation. Neuroscientist 18, 251–270. 10.1177/1073858411403316, PMID: 21677128 PMC3553600

[ref3] Andrews-Hanna J. R. Smallwood J. Spreng R. N. (2014). The default network and self-generated thought: component processes, dynamic control, and clinical relevance. Ann. N. Y. Acad. Sci. 1316, 29–52. 10.1111/nyas.12360 24502540 PMC4039623

[ref4] Andrews-Hanna J. R. Snyder A. Z. Vincent J. L. Lustig C. Head D. Raichle M. E.. (2007). Disruption of large-scale brain systems in advanced aging. Neuron 56, 924–935. 10.1016/j.neuron.2007.10.038, PMID: 18054866 PMC2709284

[ref5] Ashby W. R. (1947). Principles of the self-organising dynamic system. J. Gen. Psychol. 37, 125–128. 10.1080/00221309.1947.9918144, PMID: 20270223

[ref6] Awh E. Jonides J. (2001). Overlapping mechanisms of attention and spatial working memory. Trends Cogn. Sci. 5, 119–126. 10.1016/s1364-6613(00)01593-x, PMID: 11239812

[ref7] Ballard D. H. Hinton G. E. Sejnowski T. J. (1983). Parallel visual computation. Nature 306, 21–26. 10.1038/306021a0, PMID: 6633656

[ref8] Barber A. D. Caffo B. S. Pekar J. J. Mostofsky S. H. (2013). Developmental changes in within- and between-network connectivity between late childhood and adulthood. Neuropsychologia 51, 156–167. 10.1016/j.neuropsychologia.2012.11.011, PMID: 23174403 PMC3543510

[ref9] Barrett L. F. Simmons W. K. (2015). Interoceptive predictions in the brain. Nat. Rev. Neurosci. 16, 419–429. 10.1038/nrn3950, PMID: 26016744 PMC4731102

[ref10] Beer J. S. (2007). The default self: feeling good or being right? Trends Cogn. Sci. 11, 187–189. 10.1016/j.tics.2007.02.004, PMID: 17347027

[ref2000] Bion W. R. (1959). “Attacks on linking” in *Melanie Klein today: Developments in theory and practice*. *Volume 1: Mainly theory. 1988*. ed. Bott Spillius E. (London: Routledge).

[ref11] Bion W. R. (1962a). A theory of thinking. Int. J. Psychoanal. 43, 178–186. 13968380

[ref12] Bion W. R. (1962b). Learning from experience. London: Heinemann.

[ref13] Bion W. R. (1965). Transformations. London: Heinemann.

[ref14] Bion W. R. (1973). Elements of psycho-analysis. London: Heinemann.

[ref15] Block N. (1980). Readings in philosophy of psychology. Cambridge, MA: Harvard University Press.

[ref16] Botvinick M. M. Cohen J. D. Carter C. S. (2004). Conflict monitoring and anterior cingulate cortex: an update. Trends Cogn. Sci. 8, 539–546. 10.1016/j.tics.2004.10.003, PMID: 15556023

[ref17] Buckner R. L. (2013). The brain’s default network: origins and implications for the study of psychosis. Dialogues Clin. Neurosci. 15, 351–358. PMID: 24174906 10.31887/DCNS.2013.15.3/rbuckner PMC3811106

[ref18] Buckner R. L. Andrews-Hanna J. R. Schacter D. L. (2008). The brain’s default network: anatomy, function, and relevance to disease. Ann. N. Y. Acad. Sci. 1124, 1–38. 10.1196/annals.1440.011 18400922

[ref19] Carhart-Harris R. L. Friston K. J. (2010). The default-mode, ego-functions and free-energy: a neurobiological account of Freudian ideas. Brain 133, 1265–1283. 10.1093/brain/awq010 20194141 PMC2850580

[ref20] Carter C. S. MacDonald A. W. 3rd Ross L. L. Stenger V. A. (2001). Anterior cingulate cortex activity and impaired self-monitoring of performance in patients with schizophrenia: an event-related fMRI study. Am. J. Psychiatry 158, 1423–1428. 10.1176/appi.ajp.158.9.1423, PMID: 11532726

[ref21] Castellanos F. X. Margulies D. S. Kelly C. Uddin L. Q. Ghaffari M. Kirsch A.. (2008). Cingulate-precuneus interactions: a new locus of dysfunction in adult attention-deficit/hyperactivity disorder. Biol. Psychiatry 63, 332–337. 10.1016/j.biopsych.2007.06.025, PMID: 17888409 PMC2745053

[ref22] Chalmers D. J. (1996). The conscious mind: In search of a fundamental theory. Oxford: Oxford University Press.

[ref24] Church J. A. Fair D. A. Dosenbach N. U. Cohen A. L. Miezin F. M. Petersen S. E. (2009). Control networks in paediatric Tourette syndrome show immature and anomalous patterns of functional connectivity. Brain 132, 225–238. 10.1093/brain/awn223 18952678 PMC2638693

[ref25] Cieri F. Esposito R. (2018). Neuroaging through the lens of the resting state networks. Biomed. Res. Int. 2018:5080981. 10.1155/2018/5080981, PMID: 29568755 PMC5820564

[ref26] Cieri F. Esposito R. Cera N. Pieramico V. Tartaro A. Di Giannantonio M. (2017). Late-life depression: modifications of brain resting state activity. J. Geriatr. Psychiatry Neurol. 30, 140–150. 10.1177/0891988717700509 28355945

[ref27] Ciocca A. (2015). Storia della psicoanalisi. Bologna: Il Mulino.

[ref28] Cole M. W. Repovs G. Anticevic A. (2014). The frontoparietal control system: a central role in mental health. Neuroscientist 20, 652–664. 10.1177/1073858414525995, PMID: 24622818 PMC4162869

[ref29] Connolly J. P. (2016). Principles of organization of psychic energy within psychoanalysis: a systems theory perspective. Unpublished doctoral thesis Pretoria: University of South Africa.

[ref30] Connolly P. (2018). Expected free energy formalizes conflict underlying defense in Freudian psychoanalysis. Front. Psychol. 9:1264. 10.3389/fpsyg.2018.01264, PMID: 30072943 PMC6060308

[ref31] Corbetta M. Shulman G. L. (2002). Control of goal-directed and stimulus-driven attention in the brain. Nat. Rev. Neurosci. 3, 201–215. 10.1038/nrn755 11994752

[ref32] Craig A. D. (2002). How do you feel? Interoception: the sense of the physiological condition of the body. Nat. Rev. Neurosci. 3, 655–666. 10.1038/nrn894 12154366

[ref33] Craig A. D. (2009). How do you feel – now? The anterior insula and human awareness. Nat. Rev. Neurosci. 10, 59–70. 10.1038/nrn2555 19096369

[ref34] Craig A. D. (2010). The sentient self. Brain Struct. Funct. 214, 563–577. 10.1007/s00429-010-0248-y 20512381

[ref35] Craig A. D. (2011). Significance of the insula for the evolution of human awareness of feelings from the body. Ann. N. Y. Acad. Sci. 1225, 72–82. 10.1111/j.1749-6632.2011.05990.x 21534994

[ref36] Craver C. (2007). Explaining the brain. Univ. Oxford: Oxford Press.

[ref37] Critchley H. D. Harrison N. A. (2013). Visceral influences on brain and behavior. Neuron 77, 624–638. 10.1016/j.neuron.2013.02.008, PMID: 23439117

[ref38] Damasio A. R. (1996). The somatic marker hypothesis and the possible functions of the prefrontal cortex. Philos. Trans. R. Soc. Lond. Ser. B Biol. Sci. 351, 1413–1420. 8941953 10.1098/rstb.1996.0125

[ref39] Damoiseaux J. S. Rombouts S. A. Barkhof F. Scheltens P. Stam C. J. Smith S. M. (2006). Consistent resting-state networks across healthy subjects. Proc. Natl. Acad. Sci. USA 103, 13848–13853. 10.1073/pnas.0601417103 16945915 PMC1564249

[ref40] Dayan P. Hinton G. E. Neal R. M. (1995). The Helmholtz machine. Neural Comput. 7, 889–904. 10.1162/neco.1995.7.5.889, PMID: 7584891

[ref41] Deco G. Corbetta M. (2011). The dynamical balance of the brain at rest. Neuroscientist 17, 107–123. 10.1177/1073858409354384, PMID: 21196530 PMC4139497

[ref42] Deco G. Jirsa V. K. McIntosh A. R. (2011). Emerging concepts for the dynamical organization of resting-state activity in the brain. Nat. Rev. Neurosci. 12, 43–56. 10.1038/nrn2961, PMID: 21170073

[ref43] Di X. Biswal B. B. (2015). Characterizations of resting-state modulatory interactions in the human brain. J. Neurophysiol. 114, 2785–2796. 10.1152/jn.00893.2014, PMID: 26334022 PMC4737428

[ref44] Esposito R. Cieri F. Chiacchiaretta P. Lauriola M. Di Giannantonio M. Tartaro A.. (2018a). Modifications in resting state functional anticorrelation between default mode network and dorsal attention network: comparison among young adults, healthy elders and mild cognitive impairment patients. Brain Imaging Behav. 12, 127–141. 10.1007/s11682-017-9686-y, PMID: 28176262

[ref45] Esposito R. Cieri F. di Giannantonio M. Tartaro A. (2018b). The role of body image and self-perception in anorexia nervosa: the neuroimaging perspective. J. Neuropsychol. 12, 41–52. 10.1111/jnp.12106, PMID: 27220759

[ref46] Ferrari A. B. (1992). L’eclissi del corpo. Borla: Roma.

[ref47] Fox M. D. Raichle M. E. (2007). Spontaneous fluctuations in brain activity observed with functional magnetic resonance imaging. Nat. Rev. Neurosci. 8, 700–711. 10.1038/nrn2201, PMID: 17704812

[ref48] Fox M. D. Snyder A. Z. Zacks J. M. Raichle M. E. (2006). Coherent spontaneous activity accounts for trial-to-trial variability in human evoked brain responses. Nat. Neurosci. 9, 23–25. 10.1038/nn1616, PMID: 16341210

[ref49] Fransson P. (2005). Spontaneous low-frequency BOLD signal fluctuations: an fMRI investigation of the resting-state default mode of brain function hypothesis. Hum. Brain Mapp. 26, 15–29. 10.1002/hbm.20113, PMID: 15852468 PMC6871700

[ref600] Freud S. (1940). An outline of psychoanalysis. Std Edn. Vol. 23. London: Vintage.

[ref500] Freud S. (1911). Formulations on the two principles of mental functioning. Std Edn. Vol. 12. 218–286.

[ref50] Freud S. (1895/1963). “On the grounds for detaching a particular syndrome from neurasthenia under the description ‘anxiety neurosis’” in The standard edition of the complete psychological works of Sigmund Freud. Vol. 3, ed. Strachey J. trans. (London: The Hogarth Press).

[ref51] Freud S. (1895/1966). “Project for a scientific psychology” in The standard edition of the complete psychological works of Sigmund Freud. Vol. 1, ed. Strachey J. trans. (London: The Hogarth Press).

[ref53] Freud S. (1915). Instincts and their vicissitudes. The standard edition of the complete psychological works of Sigmund Freud, volume XIV (1914–1916): On the history of the psycho-analytic movement, papers on metapsychology and other works. 109–140.

[ref55] Freud S. (1923). “The ego and the Id” in The standard edition of the complete psychological works of Sigmund Freud: The ego and the Id and other works. Vol. 19, eds. Strachey J. Freud A. Strachey A. Tyson A., (London: Vintage), 1–66.

[ref56] Freud A. (1937). The ego and the mechanisms of defence. London: Hogarth Press and Institute of Psycho-Analysis.

[ref57] Friston K. J. (2005). A theory of cortical responses. Philos. Trans. R. Soc. Lond. Ser. B Biol. Sci. 360, 815–836. 15937014 10.1098/rstb.2005.1622 PMC1569488

[ref58] Friston K. (2009). The free-energy principle: a rough guide to the brain? Trends Cogn. Sci. 13, 293–301. 10.1016/j.tics.2009.04.005 19559644

[ref59] Friston K. (2010). The free-energy principle: a unified brain theory? Nat. Rev. Neurosci. 11, 127–138. 10.1038/nrn2787 20068583

[ref62] Friston K. Kilner J. Harrison L. (2006). A free energy principle of the brain. J. Physiol. 100, 70–87. 10.1016/j.jphysparis.2006.10.001 17097864

[ref63] Friston K. Levin M. Sengupta B. Pezzulo G. (2015a). Knowing one’s place: a free-energy approach to pattern regulation. J. R. Soc. Interface 12, 1–12. 10.1098/rsif.2014.1383 PMC4387527 25788538

[ref64] Friston K. Rigoli F. Ognibene D. Mathys C. Fitzgerald T. Pezzulo G. (2015b). Active inference and epistemic value. Cogn. Neurosci. 6, 187–214. 10.1080/17588928.2015.1020053 25689102

[ref66] Gopinath K. Krishnamurthy V. Cabanban R. Crosson B. A. (2015). Hubs of anticorrelation in high-resolution resting-state functional connectivity network architecture. Brain Connect. 5, 267–275. 10.1089/brain.2014.0323 25744222

[ref67] Gregory R. L. (1980). Perceptions as hypotheses. Philos. Trans. R. Soc. Lond. Ser. B Biol. Sci. 290, 181–197. 6106237 10.1098/rstb.1980.0090

[ref68] Greicius M. D. Krasnow B. Reiss A. L. Menon V. (2003). Functional connectivity in the resting brain: a network analysis of the default mode hypothesis. Proc. Natl. Acad. Sci. USA 100, 253–258. 10.1073/pnas.0135058100 12506194 PMC140943

[ref69] Gusnard D. A. Raichle M. E. (2001). Searching for a baseline: functional imaging and the resting human brain. Nat. Rev. Neurosci. 2, 685–694. 10.1038/35094500, PMID: 11584306

[ref700] Helmholtz H. (1866/1962). “Concerning the perceptions in general” in Treatise on physiological optics. 3rd Edn. Vol. III. ed. Southall J. trans. (New York: Dover).

[ref70] Hopkins J. (2012). “Psychoanalysis, representation and neuroscience: the Freudian unconscious and the Bayesian brain” in From the couch to the lab: Psychoanalysis, neuroscience and cognitive psychology in dialogue. eds. Fotopoulu A. Pfaff D. Conway M. (Oxford: Oxford University Press), 230–265.

[ref71] Hopkins J. (2015). “The significance of consilience: psychoanalysis, attachment, neuroscience, and evolution” in Psychoanalysis and philosophy of mind: Unconscious mentality in the 21st century. eds. Boag S. Brakel L. Talvitie V. (London: Karnac), 47–136.

[ref72] Hopkins J. (2016). Free energy and virtual reality in neuroscience and psychoanalysis: a complexity theory of dreaming and mental disorder. Front. Psychol. 7:922. 10.3389/fpsyg.2016.00922 27471478 PMC4946392

[ref73] James W. (1890). “The principles of psychology” (London: MacMillan).

[ref74] Kandel E. (1999). Biology and the future of psychoanalysis: a new intellectual framework for psychiatry revisited. Am. J. Psychiatry 156, 505–524. 10200728 10.1176/ajp.156.4.505

[ref75] Kauffman S. (1993). The origins of order: Self-organization and selection in evolution. Oxford: Oxford University Press.

[ref76] Kirchhoff M. Parr T. Palacios E. Friston K. Kiverstein J. (2018). The Markov blankets of life: autonomy, active inference and the free energy principle. J. R. Soc. Interface 15:20170792. 10.1098/rsif.2017.0792 29343629 PMC5805980

[ref77] Knill D. C. Pouget A. (2004). The Bayesian brain: the role of uncertainty in neural coding and computation. Trends Neurosci. 27, 712–719. 10.1016/j.tins.2004.10.007, PMID: 15541511

[ref78] Levin K. (1935). “Dynarnic theory of personality” in Psychology ed. Dashiell J. F. (New York and London: McGraw-Hill Book Company, Inc.).

[ref79] Livianos-Aldana L. Rojo-Moreno L. Sierra-Sanmiguel P. F. J. (2007). Gall and the phrenological movement. Am. J. Psychiatry 164:414. 10.1176/ajp.2007.164.3.414, PMID: 17329465

[ref80] Lombardi R. (2016). Metà prigioniero, metà alato. La dissociazione corpo-mente in psicoanalisi. Bollati Boringhieri: Torino.

[ref81] Lombardi R. Pola M. (2010). The body, adolescence, and psychosis. Int. J. Psychoanal. 91, 1419–1444. 10.1111/j.1745-8315.2010.00356.x 21133906

[ref82] Lurija A. R. (1976). Working brain: An introduction to neuropsychology. ISBN 046509208X (ISBN13: 9780465092086).

[ref83] Mantini D. Corbetta M. Perrucci M. G. Romani G. L. Del Gratta C. (2009). Large-scale brain networks account for sustained and transient activity during target detection. NeuroImage 44, 265–274. 10.1016/j.neuroimage.2008.08.019, PMID: 18793734 PMC5745806

[ref84] Mason M. F. Norton M. I. Van Horn J. D. Wegner D. M. Grafton S. T. Macrae C. N. (2007). Wandering minds: the default network and stimulus-independent thought. Science 315, 393–395. 10.1126/science.1131295, PMID: 17234951 PMC1821121

[ref85] McEwen B. S. (2004). “Protective and damaging effects of the mediators of stress and adaptation: allostasis and allostatic load” in Allostasis, homeostasis, and the costs of physiological adaptation. ed. Schulkin J. (Cambridge, MA: Cambridge University Press), 65–98.

[ref86] McEwen B. S. (2007). Physiology and neurobiology of stress and adaptation: central role of the brain. Physiol. Rev. 87, 873–904. 10.1152/physrev.00041.2006 17615391

[ref87] Mellor M. J. (2018). Making worlds in a waking dream: where Bion intersects Friston on the shaping and breaking of psychic reality. Front. Psychol. 9:1674. 10.3389/fpsyg.2018.01674, PMID: 30319479 PMC6171158

[ref88] Menon V. (2011). Large-scale brain networks and psychopathology: a unifying triple network model. Trends Cogn. Sci. 15, 483–506. 10.1016/j.tics.2011.08.003 21908230

[ref89] Menon V. Uddin L. Q. (2010). Saliency, switching, attention and control: a network model of insula function. Brain Struct. Funct. 214, 655–667. 10.1007/s00429-010-0262-0, PMID: 20512370 PMC2899886

[ref800] Miller J. G. (1978). Living systems. New York: McGraw-Hill.

[ref90] Molnar-Szakacs I. Uddin L. Q. (2013). Self-processing and the default mode network: interactions with the mirror neuron system. Front. Hum. Neurosci. 7:571. 10.3389/fnhum.2013.00571, PMID: 24062671 PMC3769892

[ref91] Moran L. V. Tagamets M. A. Sampath H. O’Donnell A. Stein E. A. Kochunov P.. (2013). Disruption of anterior insula modulation of large-scale brain networks in schizophrenia. Biol. Psychiatry 74, 467–474. 10.1016/j.biopsych.2013.02.029, PMID: 23623456 PMC3735654

[ref92] O’Donnell C. van Rossum M. C. (2014). Systematic analysis of the contributions of stochastic voltage gated channels to neuronal noise. Front. Comput. Neurosci. 8:105. 10.3389/fncom.2014.00105 25360105 PMC4199219

[ref93] Ortega P. A. Braun D. A. (2010). A minimum relative entropy principle for learning and acting. J. Artif. Intell. Res. 38, 475–511.

[ref94] Passow S. Specht K. Adamsen T. C. Biermann M. Brekke N. Craven A. R. (2015). Default-mode network functional connectivity is closely related to metabolic activity. Hum. Brain Mapp. 36, 2027–2038. 10.1002/hbm.22753 25644693 PMC5006878

[ref96] Phan K. L. Wager T. Taylor S. F. Liberzon I. (2002). Functional neuroanatomy of emotion: a meta-analysis of emotion activation studies in PET and fMRI. NeuroImage 16, 331–348. 10.1006/nimg.2002.1087, PMID: 12030820

[ref97] Posner M. I. (1994). Attention: the mechanisms of consciousness. 1994. Proc. Natl. Acad. Sci. USA 91, 7398–7403. 8052596 10.1073/pnas.91.16.7398 PMC44408

[ref98] Qin P. Northoff G. (2011). How is our self related to midline regions and the default-mode network? NeuroImage 57, 1221–1233. 10.1016/j.neuroimage.2011.05.028, PMID: 21609772

[ref99] Raichle M. E. MacLeod A. M. Snyder A. Z. Powers W. J. Gusnard D. A. Shulman G. L. (2001). A default mode of brain function. Proc. Natl. Acad. Sci. USA 98, 676–682. 10.1073/pnas.98.2.676 11209064 PMC14647

[ref100] Ramstead M. J. D. Badcock P. J. Friston K. J. (2018). Answering Schrödinger’s question: a free-energy formulation. Phys. Life Rev. 24, 1–16. 10.1016/j.plrev.2017.09.001 29029962 PMC5857288

[ref101] Rapaport D. (1960). The structure of psychoanalytic theory. Psychol. Issues 2, 1–158.

[ref900] Schneider F. Bermpohl F. Heinzel A. Rotte M. Walter M. Tempelmann C. (2008). The resting brain and our self: self-relatedness modulates resting state neural activity in cortical midline structures. Neuroscience 157, 120–131. 10.1016/j.neuroscience.2008.08.014 18793699

[ref103] Seeley W. W. Menon V. Schatzberg A. F. Keller J. Glover G. H. Kenna H. (2007). Dissociable Intrinsic Connectivity Networks for Salience Processing and Executive Control. J. Neurosci. 27, 2349–2356. 10.1523/JNEUROSCI.5587-06.2007 17329432 PMC2680293

[ref104] Seojung L. Kyung B. K. Jeonghun K. Jung-Hyun L. Kee N. Young-Chul J. (2014). Resting-state synchrony between anterior cingulate cortex and precuneus relates to body shape concern in anorexia nervosa and bulimia nervosa. Psychiatry Res. 221, 43–48. 10.1016/j.pscychresns.2013.11.004 24300085

[ref901] Seth A. K. Friston K. J. (2016). Active interoceptive inference and the emotional brain. Phil. Trans. R. Soc. B 371:20160007. 10.1098/rstb.2016.0007 28080966 PMC5062097

[ref106] Shulman G. L. Fiez J. A. Corbetta M. Buckner R. L. Miezin F. M. Raichle M. E.. (1997). Common blood flow changes across visual tasks: II. Decreases in cerebral cortex. J. Cogn. Neurosci. 9, 648–663. 10.1162/jocn.1997.9.5.648, PMID: 23965122

[ref107] Singer T. Critchley H. D. Preuschoff K. (2009). A common role of insula in feelings, empathy and uncertainty. Trends Cogn. Sci. 13, 334–340. 10.1016/j.tics.2009.05.001, PMID: 19643659

[ref108] Solms M. (2014). A neuropsychoanalytical approach to the hard problem of consciousness. J. Integr. Neurosci. 13, 173–185. 10.1142/S0219635214400032, PMID: 25012708

[ref109] Solms M. (2019). The hard problem of consciousness and the free energy principle. Front. Psychol. 9:2714. 10.3389/fpsyg.2018.02714 30761057 PMC6363942

[ref110] Sterling P. (2004). “Principles of allostasis: optimal design, predictive regulation, pathophysiology, and rational therapeutics” in Allostasis, homeostasis, and the costs of physiological adaptation. ed. Schulkin J. (Cambridge, MA: Cambridge University Press).

[ref111] Sterling P. Eyer J. (1988). “Allostasis: a new paradigm to explain arousal pathology” in Handbook of life stress, cognition, and health. eds. Fisher S. Reason J. (Chichester, UK: John Wiley and Sons), 629–649.

[ref902] Szpunar K. K. Watson J. M. McDermott K. B. (2007). Neural substrates of envisioning the future. Proc. Natl. Acad. Sci. USA 104, 642–647. 10.1073/pnas.0610082104 17202254 PMC1761910

[ref112] Tretter F. Löffler-Stastka H. (2018). Steps toward an integrative clinical systems psychology. Front. Psychol. 9:1616. 10.3389/fpsyg.2018.01616 30283371 PMC6157403

[ref113] Turner G. R. Spreng R. N. (2015). Prefrontal engagement and reduced default network suppression co-occur and are dynamically coupled in older adults: the default-executive coupling hypothesis of aging. J. Cogn. Neurosci. 27, 2462–2476. 10.1162/jocn_a_00869 26351864

[ref115] van den Heuvel M. P. Hulshoff Pol H. E. (2010). Exploring the brain network: a review on resting-state fMRI functional connectivity. Eur. Neuropsychopharmacol. 20, 519–534. 10.1016/j.euroneuro.2010.03.008 20471808

[ref903] von Bertalanffy L. (1967). General system theory: Foundations, development, applications. New York: George Braziller.

[ref116] von Helmholtz H. (1962). “Concerning the perceptions in general” in Treatise on physiological optics, 3rd Edn. Vol. III (translated by J. P. C. Southall 1925 Opt. Soc. Am. Section 26, reprinted New York: Dover, 1962).

[ref118] Wu J. T. Wu H. Z. Yan C. G. Chen W. X. Zhang H. Y. He Y. (2011). Aging-related changes in the default mode network and its anti-correlated networks: a resting-state fMRI study. Neurosci. Lett. 504, 62–67. 10.1016/j.neulet.2011.08.059 21925236

[ref119] Yu Y. Yang J. Ejima Y. Fukuyama H. Wu J. (2018). Asymmetric functional connectivity of the contra- and ipsilateral secondary somatosensory cortex during tactile object recognition. Front. Hum. Neurosci. 11:662. 10.3389/fnhum.2017.00662 29416506 PMC5787555

[ref120] Zepf S. (2010). Libido and psychic energy – Freud’s concepts reconsidered. Int. Forum Psychoanal. 19, 3–14. 10.1080/08037060802450753

